# Supplementary material for: The Renalase Asp37Glu polymorphism is not associated with hypertension and cardiovascular events in an urban-based prospective cohort: the Malmö Diet and cancer study
Source: BMC Med Genet. 2012 Jul 19;13:57. doi: 10.1186/1471-2350-13-57 (PMC3458972; doi:10.1186/1471-2350-13-57)
Supplement: Additional file 1 — Table S1. Association between the RNLS polymorphisms and BP according to different genetic model after excluding subjects with previous CV events, diabetes mellitus and CKD (n=4209). Table S2. Association between the RNLS polymorphisms and BP according to different genetic model after further adjustment for glucose, triglycerides, total cholesterol, high density lipoprotein cholesterol, serum cystatin C, smoking and drinking status (n=4209). Table S3. Beta coefficient and SE for interaction terms (either sex or age) with RNLS rs2576178 and rs2296545 polymorphisms and BP-related traits according to different mode of inheritance. Table S4. Association between the RNLS rs2296545 polymorphism and BP according to different genetic model in subjects with impaired kidney function. [file 1471-2350-13-57-S1.doc]

**Supplementary Table S1. Association between the *RNLS* polymorphisms and BP according to different genetic model after excluding subjects with previous CV events, diabetes mellitus and CKD (n=4209).**

|  |  |  | *RNLS* rs2576178 |  |  |  |
| --- | --- | --- | --- | --- | --- | --- |
|  | **Genetic model** | | | | | |
| **Type of blood pressure adjustment for AHT** | **Additive** | **p-value** | **Autosomal recessive** | **p-value** | **Autosomal dominant** | **p-value** |
| Crude (non adjusted) |  |  |  |  |  |  |
| SBP (mmHg) | 0.081 (0.420) | 0.85 | 0.622 (1.003) | 0.53 | -0.045 (0.533) | 0.93 |
| DBP (mmHg) | 0.224 (0.217) | 0.30 | 0.681 (0.518) | 0.19 | 0.169 (0.275) | 0.54 |
| Fixed addition |  |  |  |  |  |  |
| SBP (mmHg) | 0.168 (0.444) | 0.70 | 0.559 (1.060) | 0.60 | 0.113 (0.563) | 0.84 |
| DBP (mmHg) | 0.267 (0.229) | 0.24 | 0.650 (0.547) | 0.23 | 0.247 (0.291) | 0.39 |
| Stepped addition |  |  |  |  |  |  |
| SBP (mmHg) | 0.128 (0.444) | 0.77 | 0.521 (1.061) | 0.62 | 0.059 (0.564) | 0.92 |
| DBP (mmHg) | 0.236 (0.233) | 0.31 | 0.588 (0.557) | 0.29 | 0.215 (0.296) | 0.47 |
|  |  |  | ***RNLS* rs2296545** |  |  |  |
| **Type of blood pressure adjustment for AHT** | **Additive** | **p-value** | **Autosomal recessive** | **p-value** | **Autosomal dominant** | **p-value** |
| Crude (non adjusted) |  |  |  |  |  |  |
| SBP (mmHg) | -0.064 (0.379) | 0.87 | 0.027 (0.657) | 0.97 | -0.174 (0.586) | 0.77 |
| DBP (mmHg) | -0.190 (0.195) | 0.33 | -0.191 (0.339) | 0.57 | -0.301 (0.302) | 0.32 |
| *Fixed addition* |  |  |  |  |  |  |
| SBP (mmHg) | -0.191 (0.400) | 0.63 | 0.125 (0.694) | 0.86 | -0.358 (0.619) | 0.56 |
| DBP (mmHg) | -0.253 (0.206) | 0.22 | -0.267 (0.358) | 0.46 | -0.392 (0.319) | 0.22 |
| Stepped addition |  |  |  |  |  |  |
| SBP (mmHg) | -0.159 (0.401) | 0.69 | -0.092 (0.695) | 0.89 | -0.307 (0.620) | 0.62 |
| DBP (mmHg) | -0.233 (0.210) | 0.267 | -0.236 (0.365) | 0.52 | -0.370 (0.325) | 0.26 |

Adjustment for age, sex, BMI.

**Supplementary Table S2. Association between the *RNLS*** polymorphisms and BP according to different genetic model after further adjustment for glucose, triglycerides, total cholesterol, high density lipoprotein cholesterol, serum cystatin C, smoking and drinking status (n=4209).

|  |  |  | *RNLS* rs2576178 |  |  |  |
| --- | --- | --- | --- | --- | --- | --- |
|  | **Genetic model** | | | | | |
| **Type of blood pressure adjustment for AHT** | **Additive** | **p-value** | **Autosomal recessive** | **p-value** | **Autosomal dominant** | **p-value** |
| Crude (non adjusted) |  |  |  |  |  |  |
| SBP (mmHg) | 0.119 (0.424) | 0.78 | 1.045 (1.013) | 0.30 | -0.102 (0.538) | 0.85 |
| DBP (mmHg) | 0.257 (0.218) | 0.24 | 0.917 (0.522) | 0.08 | 0.156 (0.277) | 0.57 |
| Fixed addition |  |  |  |  |  |  |
| SBP (mmHg) | 0.221 (0.447) | 0.62 | 1.024 (1.070) | 0.34 | 0.068 (0.568) | 0.90 |
| DBP (mmHg) | 0.308 (0.230) | 0.18 | 0.906 (0.551) | 0.10 | 0.241 (0.292) | 0.41 |
| Stepped addition |  |  |  |  |  |  |
| SBP (mmHg) | 0.175 (0.448) | 0.70 | 0.980 (1.072) | 0.36 | 0.006 (0.569) | 0.99 |
| DBP (mmHg) | 0.274 (0.235) | 0.24 | 0.843 (0.561) | 0.13 | 0.203 (0.298) | 0.49 |
|  |  |  | ***RNLS* rs2296545** |  |  |  |
| **Type of blood pressure adjustment for AHT** | **Additive** | **p-value** | **Autosomal recessive** | **p-value** | **Autosomal dominant** | **p-value** |
| Crude (non adjusted) |  |  |  |  |  |  |
| SBP (mmHg) | -0.047 (0.381) | 0.90 | 0.017 (0.660) | 0.98 | -0.126 (0.590) | 0.83 |
| DBP (mmHg) | -0.159 (0.196) | 0.42 | -0.139 (0.340) | 0.68 | -0.269 (0.303) | 0.37 |
| *Fixed addition* |  |  |  |  |  |  |
| SBP (mmHg) | -0.191 (0.402) | 0.63 | -0.165 (0.697) | 0.81 | -0.327 (0.623) | 0.60 |
| DBP (mmHg) | -0.231 (0.207) | 0.26 | -0.230 (0.359) | 0.52 | -0.369 (0.320) | 0.25 |
| Stepped addition |  |  |  |  |  |  |
| SBP (mmHg) | -0.160 (0.403) | 0.69 | -0.128 (0.699) | 0.86 | -0.282 (0.624) | 0.65 |
| DBP (mmHg) | -0.212 (0.211) | 0.32 | -0.196 (0.366) | 0.59 | -0.351 (0.327) | 0.28 |

Adjustment for age, sex, BMI, glucose, triglycerides, total cholesterol, high density lipoprotein cholesterol, serum cystatin C, smoking and drinking status

**Supplementary Table S3. Beta coefficient and SE for interaction terms (either sex or age) with RNLS *rs2576178* and *rs2296545* polymorphisms and BP-related traits according to different mode of inheritance.**

| **BP related traits** | **Interaction** |  |  | **Genetic model** |  |  |  |
| --- | --- | --- | --- | --- | --- | --- | --- |
|  |  | **Additive** | **p-value†** | **Autosomal recessive** | **p-value†** | **Autosomal dominant** | **p-value†** |
|  |  |  |  | ***RNLS* rs2296545** |  |  |  |
| **SBP* (mmHg)** | **SEX** | -0.267 (0.714) | 0.71 | 0.032 (1.239) | 0.98 | -0.659 (1.103) | 0.55 |
| **DBP* (mmHg)** |  | -0.199 (0.372) | 0.59 | -0.161 (0.646) | 0.80 | -0.346 (0.575) | 0.55 |
| **Hypertension** |  | -0.026 (0.085) | 0.97 | 0.014 (0.147) | 0.93 | -0.072 (0.131) | 0.58 |
| **SBP (mmHg)** | **AGE** | 0.090 (0.031) | 0.003 | 0.100 (0.103) | 0.33 | 0.293 (0.092) | 0.001 |
| **DBP (mmHg)** |  | 0.155 (0.059) | 0.009 | 0.046 (0.054) | 0.39 | 0.178 (0.048) | <0.001 |
| **Hypertension** |  | 0.019 (0.007) | 0.006 | 0.016 (0.012) | 0.20 | 0.033 (0.011) | 0.002 |
| **SBP (mmHg)** | **BMI** | -0.023 (0.088) | 0.79 | 0.034 (0.152) | 0.82 | -0.086 (0.137) | 0.53 |
| **DBP (mmHg)** |  | 0.048 (0.046) | 0.30 | 0.057 (0.079) | 0.47 | 0.069 (0.071) | 0.33 |
| **Hypertension** |  | -0.004 (0.012) | 0.99 | 0.008 (0.020) | 0.69 | -0.018 (0.018) | 0.34 |
|  |  |  |  | ***RNLS rs2576178*** |  |  |  |
| **SBP (mmHg)** | **SEX** | -0.316 (0.787) | 0.69 | 0.840 (1.867) | 0.65 | -0.736 (1.004) | 0.46 |
| **DBP (mmHg)** |  | 0.181 (0.411) | 0.66 | 0.836 (0.975) | 0.39 | 0.058 (0.525) | 0.91 |
| **Hypertension** |  | -0.034 (0.094) | 0.99 | 0.116 (0.223) | 0.60 | -0.089 (0.119) | 0.46 |
| **SBP (mmHg)** | **AGE** | -0.001 (0.065) | 0.95 | -0.056 (0.153) | 0.71 | 0.014 (0.083) | 0.87 |
| **DBP (mmHg)** |  | -0.027 (0.034) | 0.43 | -0.086 (0.080) | 0.28 | -0.019 (0.044) | 0.67 |
| **Hypertension** |  | -0.006 (0.008) | 0.47 | -0.010 (0.018) | 0.59 | -0.006 (0.010) | 0.53 |
| **SBP (mmHg)** | **BM** | 0.126 (0.097) | 0.19 | -0.007 (0.227) | 0.98 | 0.211 (0.125) | 0.09 |
| **DBP (mmHg)** |  | 0.009 (0.051) | 0.86 | -0.149 (0.119) | 0.21 | 0.060 (0.065) | 0.36 |
| **Hypertension** |  | 0.001 (0.013) | 0.99 | -0.009 (0.030) | 0.76 | 0.004 (0.016) | 0.81 |

Adjustment for age, sex, BMI,

* BP values with “stepped addition” adjustment were considered for this analysis

† p-value for the interaction term

**Supplementary Table S4. Association between the *RNLS* *rs2296545* polymorphism and BP according to different genetic model in subjects with impaired kidney function.**

|  |  |  | **Mode of inheritance** |  |  |  |
| --- | --- | --- | --- | --- | --- | --- |
| **Type of blood pressure adjustment for AHT** | **Additive** | **p-value** | **Autosomal recessive** | **p-value** | **Autosomal dominant** | **p-value** |
|  | **eGFR_cystatin C_≤60 ml/min/1.73m^2^ (n=33)** | | | | | |
|  | **Additive** | **p-value** | **Autosomal recessive** | **p-value** | **Autosomal dominant** | **p-value** |
| **SBP* (mmHg)** | 3.437 (5.821) | 0.56 | 12.977 (11.674) | 0.28 | 0.710 (8.838) | 0.93 |
| **DBP* (mmHg)** | -0.740 (2.783) | 0.79 | 2.359 (5.661) | 0.68 | -2.977 (4.166) | 0.48 |
| **Hypertension** | -0.115 (0.757) | 0.88 | 0.690 (1.595) | 0.09 | -0.753 (1.284) | 0.47 |
|  | **eGFRcystatin C≤90 ml/min/1.73m^2^ (n=1158)** | | | | | |
| **SBP* (mmHg)** | 0.029 (0.836) | 0.97 | 0.538 (1.477) | 0.72 | -0.330 (1.271) | 0.79 |
| **DBP* (mmHg)** | -0.033 (0.441) | 0.94 | 0.083 (0.779) | 0.91 | -0.138 (0.670) | 0.84 |
| **Hypertension** | -0.002 (0.097) | 0.99 | -0.034 (0.171) | 0.97 | 0.020 (0.148) | 0.89 |

Adjustment for age, sex, BMI

* BP values with “stepped addition” adjustment were considered for this analysis
